# Temporal Shift When Comparing Contrast-Agent Concentration Curves Estimated Using Quantitative Susceptibility Mapping (QSM) and ΔR2*: The Association Between Vortex Parameters and Oxygen Extraction Fraction

**DOI:** 10.3390/tomography11040046

**Published:** 2025-04-09

**Authors:** Ronnie Wirestam, Anna Lundberg, Linda Knutsson, Emelie Lind

**Affiliations:** 1 Department of Medical Radiation Physics, Lund University, 221 85 Lund, Sweden; 2 F.M. Kirby Research Center for Functional Brain Imaging, Kennedy Krieger Institute, Baltimore, MD 21205, USA; 3 Department of Neurology, Johns Hopkins University School of Medicine, Baltimore, MD 21205, USA; 4 Department of Medical Imaging and Physiology, Skåne University Hospital, 22 185 Lund, Sweden

**Keywords:** dynamic susceptibility contrast, transverse relaxation rate, magnetic susceptibility, quantitative susceptibility mapping, hysteresis, vortex, microvasculature, oxygen extraction

## Abstract

Background: When plotting data points corresponding to the contrast-agent-induced change in transverse relaxation rate from a dynamic gradient-echo (GRE) magnetic resonance imaging (MRI) study versus a corresponding spin-echo study, a loop or vortex curve rather than a reversible line is formed. The vortex curve area is likely to reflect vessel architecture and oxygenation level. In this study, the vortex effect seen when using only GRE-based estimates, i.e., contrast-agent concentration based on GRE transverse relaxation rate and contrast-agent concentration based on quantitative susceptibility mapping (QSM), was investigated. Methods: Twenty healthy volunteers were examined using 3 T MRI. Magnitude and phase dynamic contrast-enhanced MRI (DSC-MRI) data were obtained using GRE echo-planar imaging. Vortex curves for grey-matter (GM) regions and for arterial input function (AIF) data were constructed by plotting concentration based on GRE transverse relaxation rate versus concentration based on QSM. Vortex parameters (vortex area and normalised vortex width) were compared with QSM-based whole-brain OEF estimates obtained using 3D GRE. Results: An obvious vortex effect was observed, and both GM vortex parameters showed a moderate and significant correlation with OEF (r = −0.51, *p* = 0.02). The vortex parameters for AIF data showed no significant correlation with OEF. Conclusions: GRE-based GM vortex parameters correlated significantly with whole-brain OEF. In agreement with expectations, the corresponding AIF data, representing high fractions of arterial blood, showed no significant correlation. Novel parameters, based solely on standard GRE protocols, are of relevance to investigate, considering that GRE-based DSC-MRI is very common in brain tumour applications.

## 1. Introduction

It is well known that the transverse relaxation rate R2*, measured by a gradient-echo (GRE) pulse sequence, and the transverse relaxation rate R2, measured by a spin-echo (SE) sequence, are influenced in different ways by the magnetic field inhomogeneities induced by a gadolinium contrast agent residing in the microvasculature [1]. In dynamic susceptibility-contrast magnetic resonance imaging (DSC-MRI), a temporal shift in the change in transverse relaxation rate can be observed between gradient-echo and spin-echo data, i.e., between the ΔR2* curve and the corresponding ΔR2 curve [2]. When plotting data points from a GRE dynamic study and a corresponding SE dynamic study as ΔR2* versus ΔR2 (or ΔR2^3/2^), a loop (or vortex curve), rather than a reversible line, is formed [3,4], and this phenomenon is sometimes referred to as a dynamic vascular hysteresis effect. Emblem et al. introduced the term vessel architectural imaging (VAI) to describe the concept [4]. Hohmann et al. recently performed whole-brain vascular architecture mapping for the establishment of VAI reference ranges, and the study identified, for example, distinct VAI-based region-specific microvascular profiles for cortical grey matter [5]. With regard to clinical applications, VAI and vessel size imaging have been suggested as potentially useful tools to characterise microvascular properties or to monitor the effects of treatment in, for example, brain cancer [6,7], dementia [8], and ischaemic stroke [9]. As a recent example, a large functional imaging study for brain metastases has suggested that vascular architecture is linked with the efficacy of immune checkpoint inhibitors [10].

The area and direction (either clockwise or counterclockwise) of the vascular hysteresis loop (or vortex curve) are likely to reflect, for example, vessel architecture and oxygenation level. Simulations indicated that a counterclockwise vortex behaviour is, in general terms, indicative of the presence of venous blood or venules [3,4]. For a uniform system of arterioles (fully oxygenised), capillaries, and venules, the vortex area, with a counterclockwise direction, tended to increase when the venule oxygen saturation levels decreased from 93% to 50% (Figure 3 in ref. [4]).

In a previous DSC-MRI study by our group, two versions of GRE tissue concentration curves, based on ΔR2* and quantitative susceptibility mapping (QSM), were compared and regarded to be similar in shape and to result in comparable perfusion estimates [11]. It is well established that R2* is dependent on how sources of magnetic susceptibility are oriented relative to the static magnetic field B_0_ [12,13], and it has been demonstrated that anisotropic cerebral vascular architecture causes an orientation dependence also in the estimation of cerebral blood flow (CBF) and cerebral blood volume (CBV) using DSC-MRI [14]. In 2010, Denk et al. demonstrated that phase and R2* exhibit different patterns of white-matter orientation dependence [15], and, of relevance to the present study, it was later shown that the apparent magnetic susceptibility χ, obtained using a QSM algorithm, also displays a different orientation dependence than R2* [16]. Hence, in retrospect, when considering this more recent study [16], and after performing a closer inspection of our previously analysed dataset [11], an investigation of whether a loop or vortex effect would be visible also when comparing ΔR2*- versus QSM-based concentration estimates appeared to be warranted. The relaxivity-based ΔR2* would, obviously, show the same features as in the original VAI concept, whereas the behaviour of tissue QSM data, in simplified terms assumed to quantify bulk susceptibility, is more difficult to predict based on previous studies. Since GRE-based DSC-MRI is very common in clinical brain tumour investigations (for tumour grading, monitoring of treatment response and differentiation between true progression and pseudo-progression, as well as between recurrence and radiation necrosis), exploration of novel parameters, based solely on standard GRE protocols is motivated.

In the present study, vortex curves resulting from dynamic ΔR2*-based versus dynamic QSM-based concentration estimates were analysed with respect to vortex area, normalised vortex width, and direction. The association between vortex parameters (including consideration of vortex direction) and QSM-based whole-brain OEF estimates was also investigated.

## 2. Materials and Methods

### 2.1. Subjects and Measurements

Measurements and associated post-processing were approved by the local ethics committee (The Regional Ethical Review Board in Lund), and written informed consent was obtained from each subject. In light of ethically motivated restraints related to the injection of a gadolinium contrast agent in healthy volunteers [17], the current study relies on an independent re-analysis of anonymised image data, acquired according to a previously reported data collection protocol [11,18]. Furthermore, the QSM-based OEF dataset has previously been used for purposes of comparison [19]. The current study is based on a separate scientific hypothesis with a different endpoint, employing post-processing approaches and analyses that were not available at the time of the previous publications.

Subjects, MRI measurement methodology, and QSM post-processing can only be briefly outlined in this short report, but have previously been described in detail [11,19]. Twenty healthy volunteers (ten females and ten males in two age groups of equal size, 25-34 years old and 51–84 years old) were examined on two occasions (referred to as visit 1 and visit 2), within a time interval of 7–20 days. The volunteers adhered to their usual pattern of food and caffeine intake during the days of examination, and all measurements took place during the daytime. Mean values of the estimates from the two visits are reported in the present study. (For one volunteer, dynamic QSM data were unavailable due to technical problems, and the reported results for this individual are thus based on only one measurement.)

MRI measurements were performed using a 3 T MRI unit (Philips Achieva, Philips Healthcare, Best, The Netherlands). Gadolinium contrast agent (0.1 mmol/kg body weight, 5 mL/s, Dotarem, Guerbet, Paris, France) was injected. Dynamic magnitude and phase DSC-MRI data were obtained using single-shot GRE echo-planar imaging (EPI) with temporal resolution 1.24 s, field of view (FOV) 220 × 220 mm^2^, image matrix 128 × 128, slice thickness 5 mm, 20 slices, flip angle (FA) 60°, echo time (TE) 29 ms, and SENSE factor 2.5. For QSM-based OEF estimation, magnitude and phase images were acquired using 3D GRE with flow compensation: 50 axial slices orthogonal to the external magnetic field, spatial resolution 0.98 × 0.98 × 1.15 ${mm}^{3}$, FOV = 220 × 220 ${mm}^{2}$, repetition time (TR) = 45 ms, TE_1_ = 20 ms, TE_2_ =40 ms, FA = 20°, and bandwidth = 218 Hz/pixel. Phase images obtained with an echo time of 40 ms were not used in the analysis due to severe aliasing effects.

### 2.2. Image Processing and Data Analysis

#### 2.2.1. Quantitative Susceptibility Mapping

QSM maps [20,21,22] from 3D-GRE and dynamic GRE-EPI data were reconstructed using the morphology-enabled dipole inversion (MEDI) toolbox (Cornell University, https://pre.weill.cornell.edu/mri/pages/qsm.html, accessed on 6 April 2025) [20,23,24,25] with background field removal using projection onto dipole fields (PDF) [26]. The regularisation parameter λ was set to 800 for 3D-GRE data and 300 for GRE-EPI data. For dynamic GRE-EPI data, tissue χ estimates were obtained using cerebrospinal fluid (CSF) as a reference region [27].

For assessment of the robustness of the GRE-based vortex concept to changes in QSM reconstruction settings, QSM maps for DSC-MRI data from one measurement were reconstructed using five different λ settings (i.e., 100, 300, 1000, 3000, and 5000).

#### 2.2.2. Oxygen Extraction Fraction

Whole-brain OEF was calculated according to Equation (1) [28], assuming that the oxygen saturation level measured in the superior sagittal sinus represents an accurate estimate of the whole-brain average OEF:

(1) $$OEF=\frac{∆\chi }{∆\chi_{do}\cdot Hct}$$

where ∆χ is the susceptibility difference between the surrounding tissue (assumed to represent arterial blood) and the venous vessel, $∆\chi_{do}=0.193\cdot 4\pi$ ppm (in SI units) is the susceptibility difference per unit haematocrit between fully deoxygenated and fully oxygenated blood [29]. Hct is the fractional haematocrit, assumed to be 0.42 for males and 0.40 for females [30]. Voxels representing venous blood in the superior sagittal sinus and surrounding tissue were selected using a previously published procedure [31].

#### 2.2.3. Vortex Curves from Dynamic GRE Data

Tissue ΔR2* and χ were converted to concentration *C* of contrast agent (CA) using

(2) $${C(t)}_{\Delta R2^{*}}=\frac{\Delta R2^{*}(t)}{r2^{*}}$$

and

(3) $${C(t)}_{QSM}=\frac{\chi (t)}{\chi_{mol}}$$

where the transverse tissue relaxivity r2* was 85 s^−1^mM^−1^ for tissue [27] and 89 s^−1^mM^−1^ for arterial input function (AIF) data [11], and $\chi_{mol}$ was 308 ppm/M [32]. Vortex curves were constructed by plotting *C*(*t*)_Δ*R*2*_ versus *C*(*t*)*_QSM_*. Note that vortex loops can exhibit intersection points that divide the loop into segments. Grey-matter (GM) vortex curves were based on data from regions of interest extracted by segmentation, using ‘*new segment*’ in SPM8, according to a previously described approach [11].

In order to compare with a dataset not assumed to reflect tissue oxygen extraction, vortex curves for DSC-MRI AIF voxel data (not previously used in ref. [11]) were also acquired and analysed. The initial selection of potential AIF pixel candidates was made automatically, in middle cerebral artery (MCA) branches, based on the shape, width, and magnitude of the curve, as well as on the arrival time of the CA, using locally developed perfusion software [33]. A visual inspection of the automatically selected time curves was subsequently performed. Only curves showing an acceptable shape (with a distinct peak and positive values in the CA steady-state period) in both $\Delta R2^{*}$-based and QSM-based images were included in the analysis.

**Figure 1:**
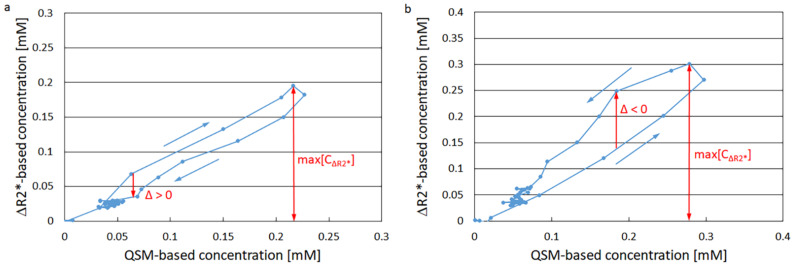
Representative examples of registered grey-matter tissue vortex curves (in blue), based on contrast-agent concentration estimates obtained using ΔR2* and quantitative susceptibility mapping (QSM). The curves also illustrate the principle for extracting values of Λ = Δ/max[C_ΔR2*_]. (**a**) Vortex curve showing clockwise direction (with Δ > 0). (**b**) Vortex curve showing counterclockwise direction (with Δ < 0).

Vortex areas were measured using a standard software tool for delineating irregular shapes and were displayed in arbitrary units (Adobe Photoshop, version 21.0.3 20200115.r.91). Vortex areas and vortex area segments corresponding to counterclockwise direction were assigned negative values. In analogy to the work by Xu et al. [3], the parameter Λ was also introduced to characterise the vortex loop:

(4) $$\Lambda =\frac{\Delta }{{\max\;[C}_{\Delta R2^{*}}]}$$

where max[C_ΔR2*_] is the maximal value of C_Δ*R*2*_ and Δ is the maximal distance from the ascending branch to the descending branch. Δ was assigned a positive value when the vortex direction at the point of maximal distance was clockwise and a negative value when it was counterclockwise (cf. Figure 1).

Vortex curves based on AIF data from one measurement, obtained with different λ values in the QSM reconstruction, were constructed and mutually compared using visual inspection.

#### 2.2.4. Statistics

The associations between vortex parameters (area and Λ) and OEF and between vortex parameters and age were assessed by linear regression and correlation analysis. For slope values, a 95% confidence interval (CI) was calculated. Under the null hypothesis that the population correlation coefficient equals zero, a *p*-value stating the probability that one would have found the current result if the correlation coefficient were in fact zero was obtained (significance level α = 0.05). The assumptions of linear regression were assessed by testing for a linear relationship, testing for normal distribution of residuals, and by visual inspection of residuals with respect to independence and homoscedasticity. A common classification of the strength of the observed associations was applied [34]: for absolute values of the Pearson correlation coefficient r, <0.20 was regarded as a very weak correlation, a range of 0.2–0.39 as weak, a range of 0.40–0.59 as moderate, a range of 0.60–0.79 as strong, and ≥0.8 as very strong.

Test–retest analyses of available vortex area and Λ data were performed using Bland–Altman plots.

## 3. Results

### 3.1. Vortex Curves

**Figure 2:**
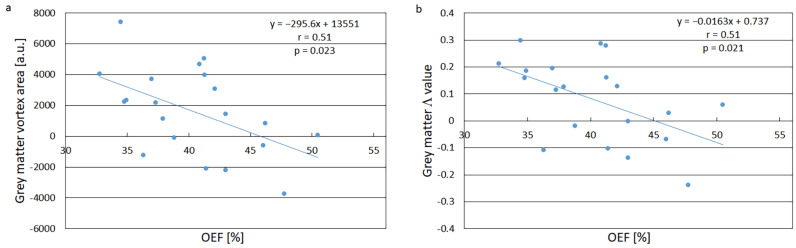
(**a**) Relationship between grey-matter tissue vortex area and whole-brain average oxygen extraction fraction (OEF). The 95% CI of the slope was [–544.6, –46.7]. (**b**) Relationship between grey-matter tissue vortex Λ value and whole-brain average OEF. The 95% CI of the slope was [–0.0299, –0.0028]. The solid lines in (**a**,**b**) are the results of linear regression analyses.

A representative example of the segmented GM region in one volunteer is displayed in Supplementary Figure S1. Representative examples of registered GM tissue vortex curves are shown in Figure 1, where Figure 1a illustrates a clockwise direction and Figure 1b shows a counterclockwise direction. The relationship between GM vortex area and whole-brain OEF is displayed in Figure 2a and the relationship between GM vortex Λ value and whole-brain OEF is given in Figure 2b. The association between GM vortex area and OEF showed a correlation coefficient of r = −0.51 (*p* = 0.023) and the association between GM Λ and OEF exhibited a correlation coefficient of r = −0.51 (*p* = 0.021). The use of a linear regression model was justified in these cases, according to the analysis reported in Supplementary Material S1. Bland–Altman plots of the GM vortex area estimates and the GM Λ estimates are shown in Supplementary Figure S2.

The AIF data vortex parameters, area and Λ, both displayed a weak and non-significant correlation with whole-brain OEF (|r| < 0.4, *p* > 0.1). AIF data vortex curves from one measurement, acquired with different λ values, are displayed in Supplementary Figure S3.

### 3.2. Age Dependence of the Vortex Parameters

**Figure 3:**
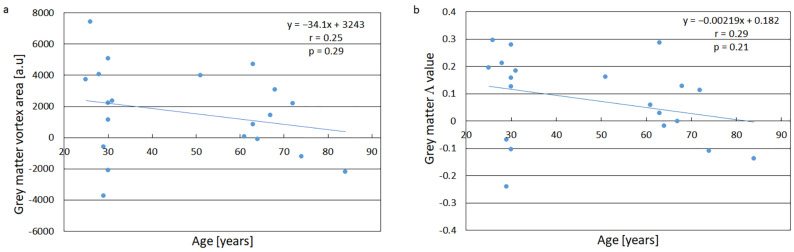
(**a**) Relationship between grey-matter tissue vortex area and age. The 95% CI of the slope was [–100.3, 32.0]. (**b**) Relationship between grey-matter tissue Λ value and age. The 95% CI of the slope was [–0.00576, 0.00138]. The solid lines in (**a**,**b**) are the results of linear regression analyses. Note that the hypothesis that there is no linear relationship could not be rejected.

The healthy volunteers were recruited to represent a large age interval, and the relationships between tissue vortex parameters and age are shown in Figure 3. For both area and Λ, the trendlines, included for completeness, showed a trend of decreasing vortex parameter (corresponding to increasing OEF) with increasing age, although the hypothesis that the correlation coefficient was equal to zero could not be rejected, neither for vortex area nor for Λ. Hence, the use of a linear regression model was not formally justified in these cases (Supplementary Material S1). For the AIF data, no discernible age trends were observed, neither for the vortex area nor for Λ (r < 0.05).

## 4. Discussion

This study should be viewed as an initial empirical exploration of whether the combination of dynamic ΔR2*-based and χ-based CA concentration data, in the formation of vortex curves, may provide useful tissue information, particularly with regard to oxygenation. An advantage of such an approach is that ΔR2* and QSM data are obtained simultaneously using the magnitude and phase, respectively, of a standard GRE image acquisition. This is of some relevance, as Emblem et al. specifically pointed out that vessel-calibre MRI, at that time, had exhibited limited accessibility to the general clinical community, partly due to the complex image acquisition process, necessitating a combination of GRE and SE protocols [35].

In general, the robustness of available QSM reconstruction procedures is still not optimal. For the specific dataset analysed in this study, the vulnerability to any fluctuations in QSM reconstruction output was, most likely, mitigated by the use of the average value from two separate measurements (cf. Supplementary Figure S2). The issue of whether the proposed GRE vortex concept shows sufficient robustness to changes in the settings of the QSM reconstruction algorithm was assessed by comparing QSM data with five different λ values. In this analysis, AIF curves were used instead of tissue curves, because the AIFs (showing sharp and narrow peaks and being based on fewer voxels than tissue ROIs) were assumed to represent a more challenging situation than the corresponding tissue data. Obviously, the resulting vortices were not identical for the five different λ values, but the vortex effect, as well as all principal properties of the vortex (overall shape, direction, intersection), were preserved over a large range of λ values (Supplementary Figure S3).

The main finding of this study is that both vortex curve area and Λ for GM (with counterclockwise direction represented by negative area and Δ values) showed a moderate and significant negative correlation with QSM-based whole-brain OEF. Indices reflecting OEF are of relevance because, despite the vital importance of oxygen, the oxygen reserves in tissue are limited, and maintained cerebral oxygen metabolism relies upon an intricate balance between CBF and OEF. A decrease in CBF is normally accompanied by a compensatory increase in OEF to ensure normal neuronal activity. Lasting brain damage may occur if the oxygen supply, in spite of the elevated OEF, becomes insufficient to uphold the required oxygen metabolism [36].

Only GM data were included in the assessments of the vortex area and Λ, in an attempt to isolate the effects of varying oxygen saturation levels. The use of spatially averaged white-matter data would have been suboptimal because the orientation dependence of R2* and the vascular anisotropy are likely to be more pronounced in white matter than in GM [37,38]. If the white matter had been included, it is likely that the results had been confounded by effects caused by white-matter microstructural orientations. These effects are indeed important, in their own right, and relevant to address [39], but contributions from white-matter microstructural orientation would, in the present study, potentially have hampered an unmitigated comparison with the OEF estimates. As previously reported [11], QSM-based concentration–time curves in some white-matter voxels tended to show an artefactual decrease in concentration during the contrast-agent bolus passage, and this served as a related, more pragmatic reason, for not including white matter in the present analysis. A potential drawback with this approach is that the employed OEF estimates represent the whole-brain average, but it is, on the other hand, well established that OEF in healthy volunteers, measured in a resting state, shows a striking spatial uniformity, despite considerable variation in resting oxygen metabolism and CBF between grey and white matter [40]. Hence, whole-brain OEF is most likely to be representative of the conditions in GM.

With regard to the relationships between vortex parameters and OEF, it should also be considered that correlation coefficients, in general, are to be interpreted with caution. In a healthy population, the OEF range among individuals is rather narrow, and the correlation coefficient tends, generally, to become lower when the interval of observations is small. It is thus encouraging that we were able to observe a significant correlation also within the limited OEF range of healthy volunteers. The AIF data were assumed to represent a much higher fraction of fully oxygenated arterial blood, not likely to vary markedly among the subjects, and the lack of AIF vortex parameter correlation with the whole-brain OEF estimates was thus in agreement with expectations.

The vortex parameters tended to decrease with age (Figure 3), but the observed relationships did not show statistically significant correlations, neither for vortex area nor for Λ, and should thus not be overinterpreted. However, this observation does, at least, not contradict previously reported patterns of OEF age dependence. For example, the comprehensive positron emission tomography study by Leenders et al. reported an OEF increase of 0.13 percentage points/year in healthy volunteers [41], which is qualitatively consistent with a decreasing vortex area and decreasing Λ. For the AIF vortex parameter data, no age-related trends were observed (r < 0.05), which was also in line with expectations.

Important future investigations, to confirm and validate the current empirical findings, include the assessment of regional variations and application to different pathological conditions (e.g., brain cancer, stroke, and dementia), as well as the development of an appropriate theoretical model and a corresponding simulation framework. Although a deeper theory- or model-based interpretation of the current experimental findings was beyond the scope of the present study, it is still interesting to note that the results show some degree of resemblance to the original VAI concept. With ΔR2*-based data placed on the y axis, as in the present study, the original VAI simulations predicted that the presence of venous blood or venules, i.e., compartments with low oxygen saturation level, tended to result in counterclockwise direction [3,4], and it was also shown that the counterclockwise vortex area increased (i.e., became more negative) when the oxygen saturation levels decreased within a physiologically reasonable interval [4]. Similarly, the present observations showed a negative correlation between tissue vortex area and OEF (where high OEF corresponds to low venous oxygen saturation). The original GRE-versus-SE VAI concept has recently proven useful in, for example, differentiation between early tumour progression and pseudo-progression in glioblastoma [7], and, bearing in mind that GRE-based DSC-MRI is very common in brain tumour applications, additional parameters, based solely on standard GRE protocols, may be of relevance to consider.

## 5. Conclusions

GRE-based vortex parameters (area and Λ), extracted from dynamic ΔR2*-based versus QSM-based GM contrast-agent concentration data, showed a moderate and significant correlation with QSM-based whole-brain OEF. In agreement with expectations, contrast-agent concentration data representing high fractions of arterial blood did not result in any significant correlation with OEF.

## Data Availability

Dataset available on request from the corresponding author.

## Supplementary Materials

The following supporting information can be downloaded at: https://www.mdpi.com/article/10.3390/tomography11040046/s1, Figure S1: A representative example of the segmented GM region in one volunteer, obtained using ‘*new segment*’ in SPM8; Material S1: Assessment of the assumptions of linear regression; Figure S2: Bland–Altman plots comparing DSC-MRI data from visit 1 and visit 2; Figure S3: Vortex curves based on arterial input function (AIF) data from one measurement, obtained using five different values of the regularization parameter λ in the MEDI QSM reconstruction.
