# Supplementary material for: Temporal Shift When Comparing Contrast-Agent Concentration Curves Estimated Using Quantitative Susceptibility Mapping (QSM) and ΔR2*: The Association Between Vortex Parameters and Oxygen Extraction Fraction
Source: Tomography. 2025 Apr 9;11(4):46. doi: 10.3390/tomography11040046 (PMC12031548; doi:10.3390/tomography11040046)

**Supplementary Material**

Figure S1. A representative example of the segmented GM region in one volunteer, obtained using '*new segment*' in SPM8.

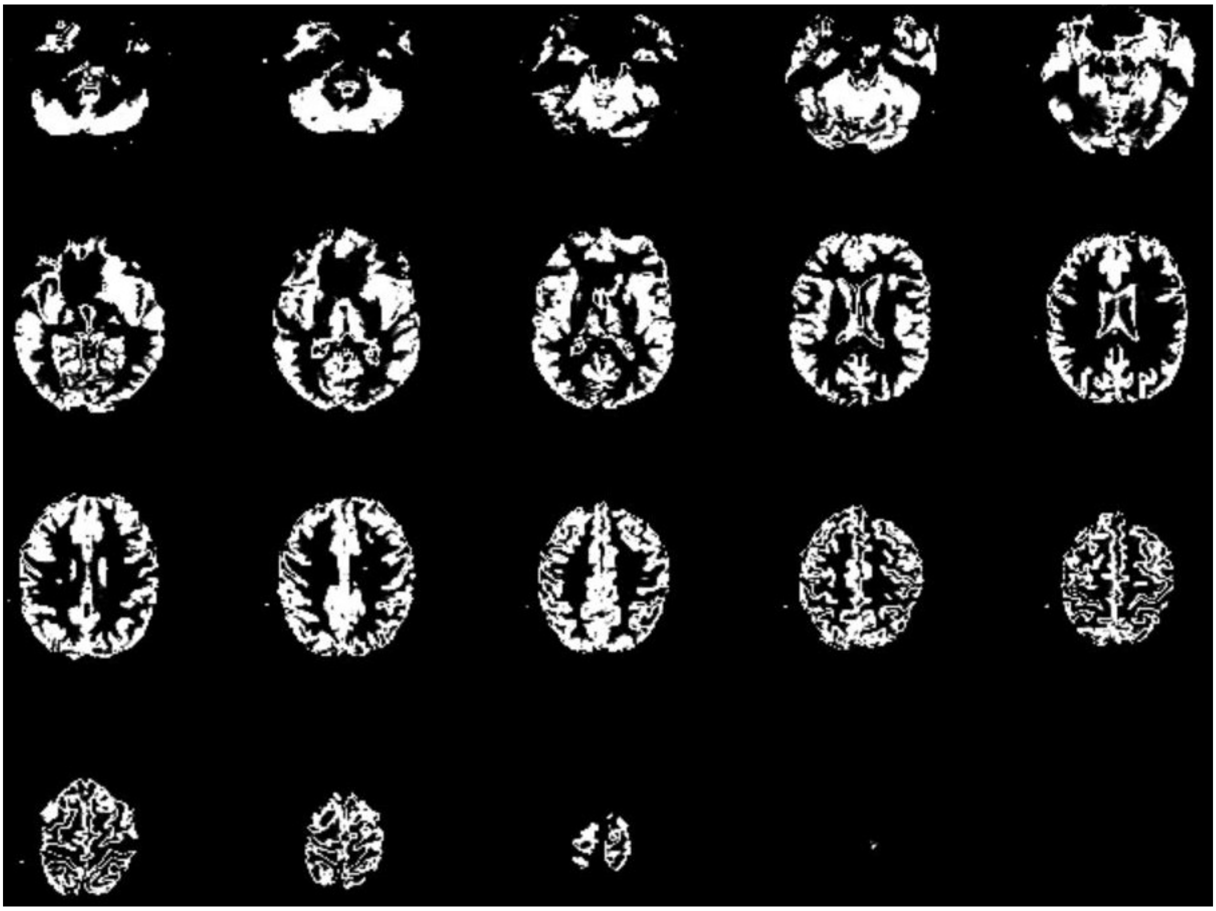

Supplement: Supplementary file 1 [file tomography-11-00046-s001.zip › Supplementary Figure S1.pdf]
