# Supplementary material for: Temporal Shift When Comparing Contrast-Agent Concentration Curves Estimated Using Quantitative Susceptibility Mapping (QSM) and ΔR2*: The Association Between Vortex Parameters and Oxygen Extraction Fraction
Source: Tomography. 2025 Apr 9;11(4):46. doi: 10.3390/tomography11040046 (PMC12031548; doi:10.3390/tomography11040046)

Supplementary Material

Figure S3. Vortex curves based on arterial input function (AIF) data from one measurement, obtained using five different values of the regularization parameter λ in the MEDI QSM reconstruction.

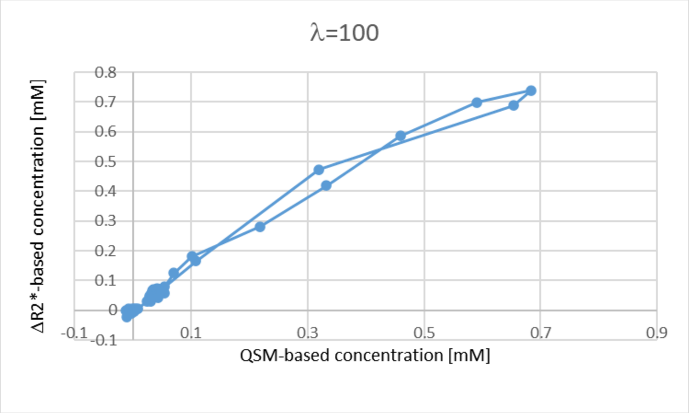

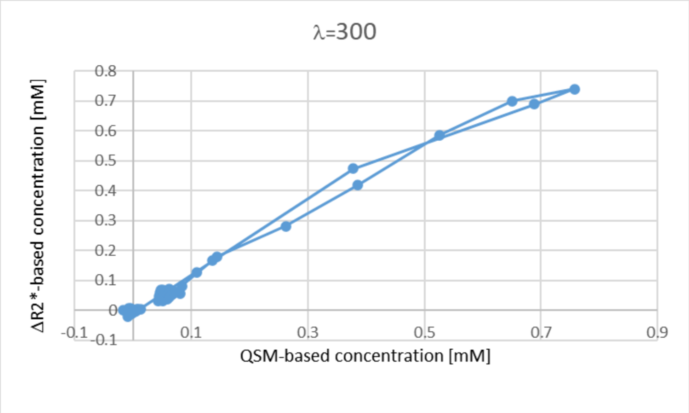

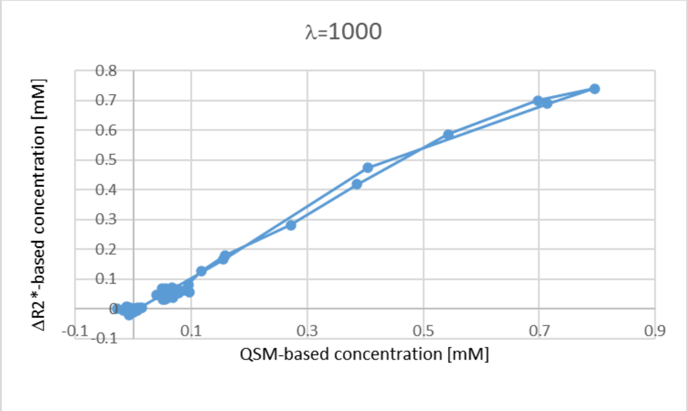

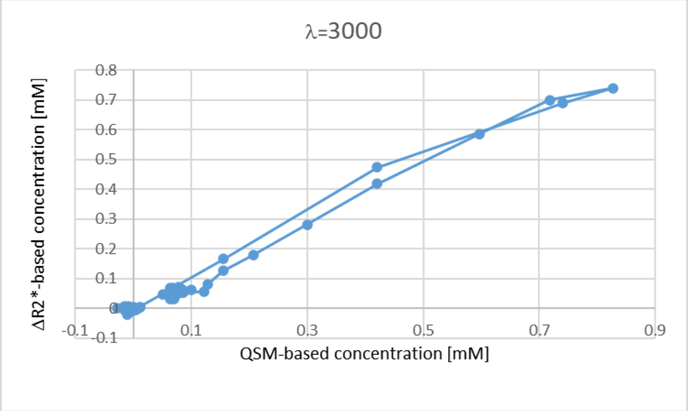

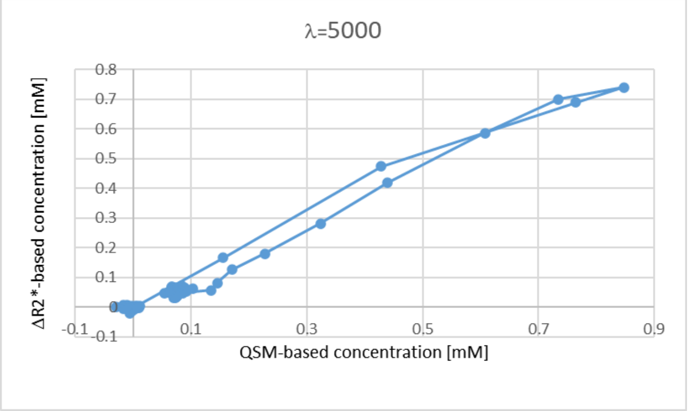

Supplement: Supplementary file 1 [file tomography-11-00046-s001.zip › Supplementary Figure S3.pdf]
