# Supplementary material for: Temporal Shift When Comparing Contrast-Agent Concentration Curves Estimated Using Quantitative Susceptibility Mapping (QSM) and ΔR2*: The Association Between Vortex Parameters and Oxygen Extraction Fraction
Source: Tomography. 2025 Apr 9;11(4):46. doi: 10.3390/tomography11040046 (PMC12031548; doi:10.3390/tomography11040046)
Supplement: Supplementary file 1 [file tomography-11-00046-s001.zip › Supplementary Material S1.pdf]

Supplementary Material S1

Assessment of the assumptions of linear regression

The results displayed below were obtained using MedCalc® Statistical Software version 23.1.3 (MedCalc Software Ltd, Ostend, Belgium; https://www.medcalc.org; 2025)

Methods

Testing for linearity

Analysis of variance: The total variation in the dependent variable is divided into two components, one which can be attributed to the regression model (referred to as ”Regression”) and one which cannot be attributed to the regression model (referred to as ”Residual”). If the significance level for the F-test is less than 0.05, the hypothesis that there is no linear relationship can be rejected.

Testing for normal distribution of residuals

The Shapiro-Wilk test was applied. If P is higher than 0.05, data are assumed to have a normal distribution, and the conclusion ‘*Accept normality*’ is shown. If P is less than 0.05, the conclusion ‘*Reject normality*’ is shown.

Results

Figure 2a: Vortex area versus OEF

Testing for linearity: Analysis of variance

Source	DF	Sum of Squares	Mean Square
Regression	1	38640237.22	38640237.22
Residual	18	111710679.00	6206148.83

F-ratio	6.23
Significance level	P=0.0225

Conclusion: The hypothesis that there is no linear relationship can be rejected.

Testing for normal distribution of residuals

Shapiro-Wilk test for Normal distribution	W=0.9674 accept Normality (P=0.6990)

Conclusion: Data are assumed to have a normal distribution.

Residuals

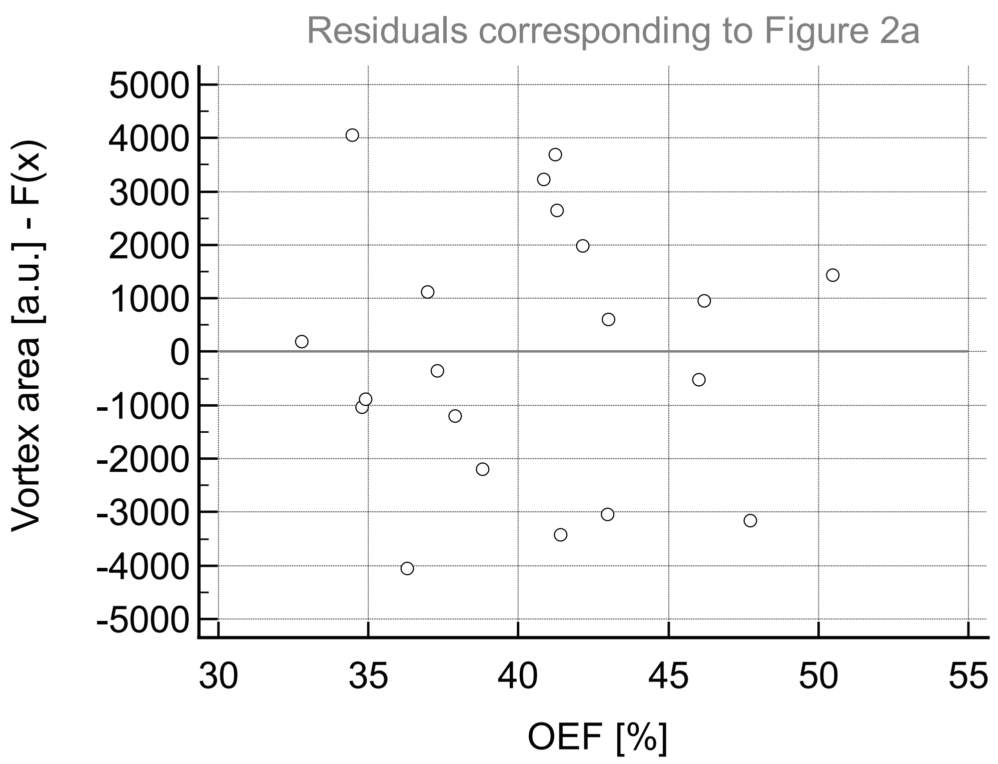

Conclusion: Visual inspection indicates that the assumptions of independence and homoscedasticity are not violated.

Figure 2b: Λ versus OEF

Testing for linearity: Analysis of variance

Source	DF	Sum of Squares	Mean Square
Regression	1	0.1180	0.1180
Residual	18	0.3313	0.01841

F-ratio	6.4098
Significance level	P=0.0209

Conclusion: The hypothesis that there is no linear relationship can be rejected.

Testing for normal distribution of residuals

Shapiro-Wilk test for Normal distribution	W=0.9701 accept Normality (P=0.7562)

Conclusion: Data are assumed to have a normal distribution.

Residuals

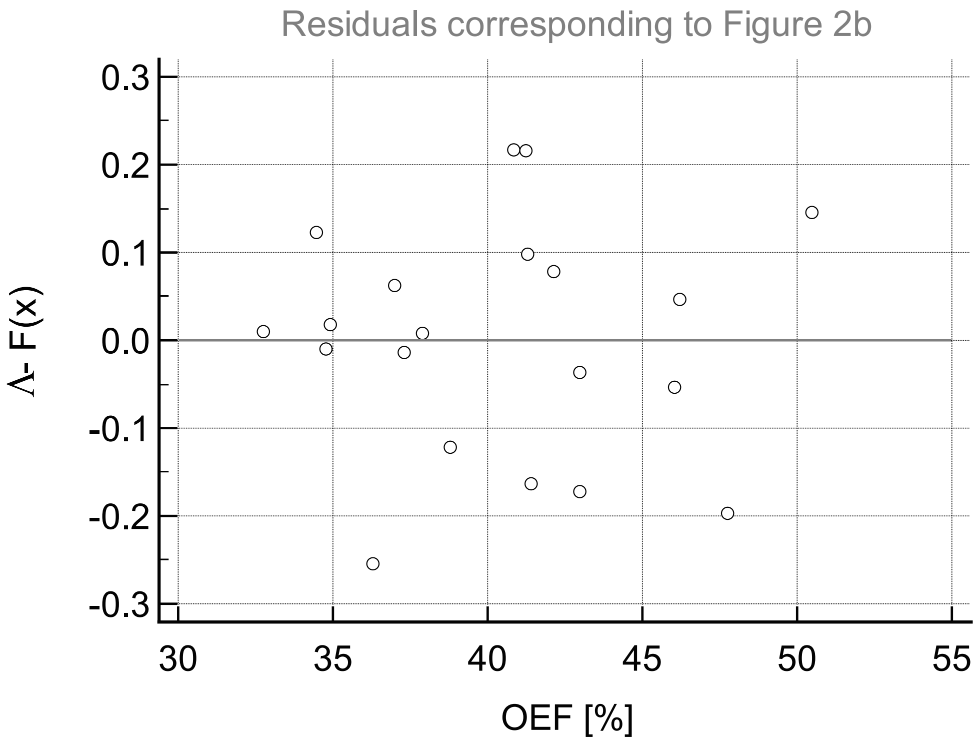

Conclusion: Visual inspection indicates that the assumptions of independence and homoscedasticity are not violated.

Figure 3a: Vortex area versus age

Testing for linearity: Analysis of variance

Source	DF	Sum of Squares	Mean Square
Regression	1	9208820.56	9208820.56
Residual	18	141142095.64	7841227.54

F-ratio	1.17
Significance level	P=0.2928

Conclusion: The hypothesis that there is no linear relationship can not be rejected.

Testing for normal distribution of residuals

Shapiro-Wilk test for Normal distribution	W=0.9893 accept Normality (P=0.9972)

Conclusion: Data are assumed to have a normal distribution.

Residuals

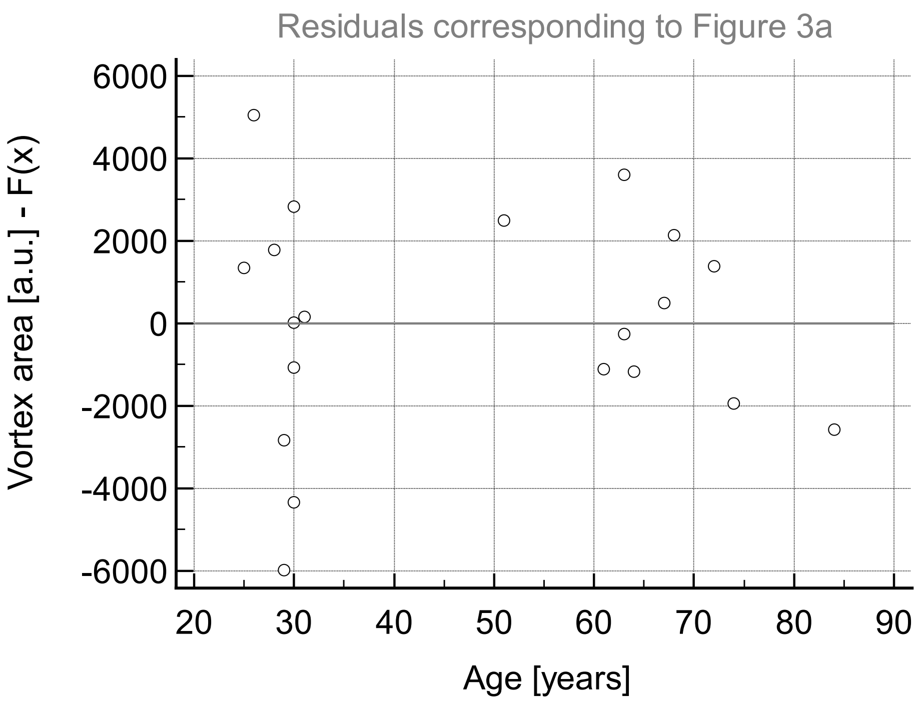

Conclusion: Visual inspection indicates that the assumptions of independence and homoscedasticity are not violated.

Figure 3b: Λ versus age

Testing for linearity: Analysis of variance

Source	DF	Sum of Squares	Mean Square
Regression	1	0.03801	0.03801
Residual	18	0.4113	0.02285

F-ratio	1.6635
Significance level	P=0.2135

Conclusion: The hypothesis that there is no linear relationship can not be rejected.

Testing for normal distribution of residuals

Shapiro-Wilk test for Normal distribution	W=0.9545 accept Normality (P=0.4401)

Conclusion: Data are assumed to have a normal distribution.

Residuals

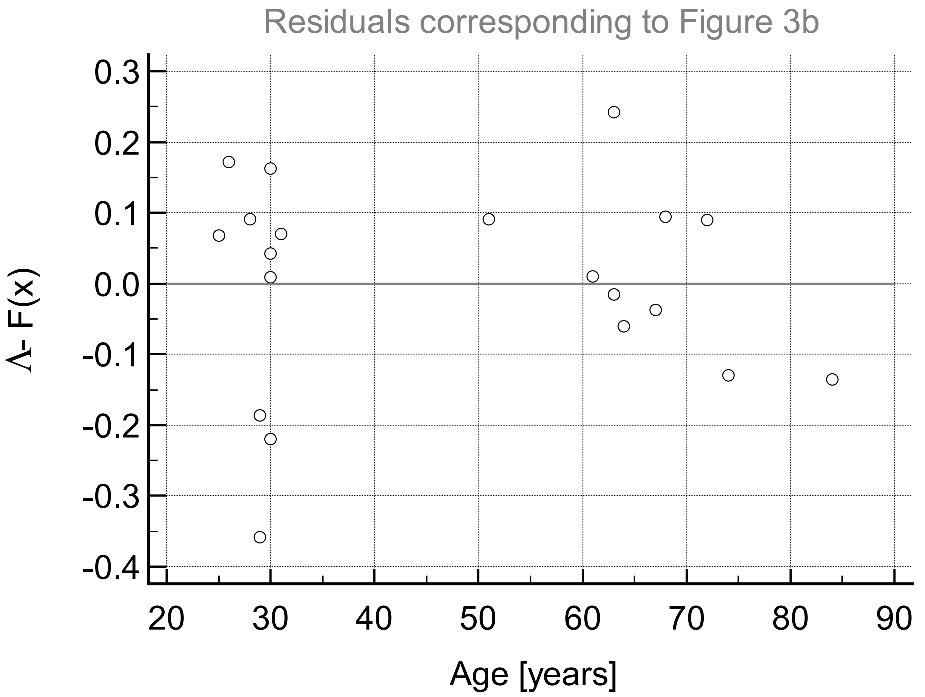

Conclusion: Visual inspection indicates that the assumptions of independence and homoscedasticity are not violated.
